# Genotype-Driven Pathogenesis of Atrial Fibrillation in Hypertrophic Cardiomyopathy: The Case of Different *TNNT2* Mutations

**DOI:** 10.3389/fphys.2022.864547

**Published:** 2022-04-19

**Authors:** Josè Manuel Pioner, Giulia Vitale, Francesca Gentile, Beatrice Scellini, Nicoletta Piroddi, Elisabetta Cerbai, Iacopo Olivotto, Jil Tardiff, Raffaele Coppini, Chiara Tesi, Corrado Poggesi, Cecilia Ferrantini

**Affiliations:** ^1^ Department of Biology, University of Florence, Florence, Italy; ^2^ Department of Experimental and Clinical Medicine, University of Florence, Florence, Italy; ^3^ Department NeuroFarBa, University of Florence, Florence, Italy; ^4^ Department of Medicine and Biomedical Engineering, University of Arizona, Tucson, AZ, United States

**Keywords:** hypertrophic cardiomyopathy, atrial myopathy, atrial fibrillation, sarcomere mechanics, sarcomere energetics, excitation-contraction coupling, cardiac troponin T

## Abstract

Atrial dilation and atrial fibrillation (AF) are common in Hypertrophic CardioMyopathy (HCM) patients and associated with a worsening of prognosis. The pathogenesis of atrial myopathy in HCM remains poorly investigated and no specific association with genotype has been identified. By re-analysis of our cohort of thin-filament HCM patients (Coppini et al. 2014) AF was identified in 10% of patients with sporadic mutations in the cardiac Troponin T gene (*TNNT2*), while AF occurrence was much higher (25–75%) in patients carrying specific “hot-spot” *TNNT2* mutations. To determine the molecular basis of arrhythmia occurrence, two HCM mouse models expressing human *TNNT2* variants (a “hot-spot” one, R92Q, and a “sporadic” one, E163R) were selected according to the different pathophysiological pathways previously demonstrated in ventricular tissue. Echocardiography studies showed a significant left atrial dilation in both models, but more pronounced in the R92Q. In E163R atrial trabeculae, in line with what previously observed in ventricular preparations, the energy cost of tension generation was markedly increased. However, no changes of twitch amplitude and kinetics were observed, and there was no atrial arrhythmic propensity. R92Q atrial trabeculae, instead, displayed normal ATP consumption but markedly increased myofilament calcium sensitivity, as previously observed in ventricular preparations. This was associated with reduced inotropic reserve and slower kinetics of twitch contractions and, importantly, with an increased occurrence of spontaneous beats and triggered contractions that represent an intrinsic arrhythmogenic mechanism promoting AF. The association of specific *TNNT2* mutations with AF occurrence depends on the mutation-driven pathomechanism (i.e., increased atrial myofilament calcium sensitivity rather than increased myofilament tension cost) and may influence the individual response to treatment.

## Introduction

Although Hypertrophic CardioMyopathy (HCM) is mostly manifested as a disease of the left ventricle (LV), atria are often involved. In a community-based HCM population, atrial fibrillation (AF) is extremely common, with a 22% prevalence over 9 years, and is associated with an increased risk of heart failure-related mortality, stroke and severe functional disability, particularly in patients with LV outflow obstruction (Olivotto et al., 2008). The genes most frequently responsible for HCM (i.e. *MYH7* encoding for β-myosin heavy chains, *MYBPC3* encoding for myosin binding protein C and the gene encoding for the thin filament protein troponin T (*TNNT2*) (Olivotto et al., 2008; Biagini et al., 2014)) are not equally and uniformly expressed in the four cardiac chambers (Piroddi et al., 2007; Belus et al., 2010)). For instance, the β-myosin heavy chain (β-MHC:*MYH7*) isoform, target of HCM-related mutations, is poorly expressed in the atria (Walklate et al., 2021), while cardiac Troponin T (cTnT) is present in both chambers with the same isoform. cTnT, in particular, is a protein closely associated to the motor function and its calcium regulation. A few case reports describing the clinical phenotype of families with severe *TNNT2*-associated HCM highlighted a high incidence of ventricular and atrial arrhythmias, including AF, despite relatively mild ventricular hypertrophy (Tardiff, 2005). These anecdotal reports suggest that mutations in specific *TNNT2* gene hot-spots may trigger sarcomere-driven mechanisms (e.g., association with increased myofilament calcium sensitivity and changes of intracellular calcium buffering) that create an arrhythmogenic substrate in both atrial and ventricular chambers (Tardiff et al., 1999).

In 2014 (Coppini et al., 2014) we assessed the clinical features and outcomes in a large cohort of patients with HCM associated with thin-filament mutations. All participants were unrelated index patients, with *TNNT2* defects being the most common (53% of the thin-filament cohort). Other thin-filament genes were *TNNI3* (30%), *TPM1* (9%), and *ACTC* (8%). None carried double thin-filament mutations. Patients carrying mutations in the most represented genes, *TNNT2* and *TNNI3*, showed remarkably similar clinical features and outcome profile compared to the thick-filament: less prominent and atypically distributed LV hypertrophy, increased LV fibrosis, higher likelihood of adverse LV remodeling leading to functional deterioration, and more frequent occurrence of triphasic LV filling, reflecting profound diastolic dysfunction. Importantly, the occurrence of ventricular and atrial arrhythmias (particularly AF) in the thin-filament and thick-filament genes appears comparable. Of note, in the thin-filament group AF was most frequently treated in an aggressive way through chateher ablation. In this study no internal comparison was made among different thin-filament genes and specific mutations in a given gene.

A mutation-specific atrial remodeling in HCM was described in cTnT mutant mice carrying different mutations in the same spot of *TNNT2* (R92W, R92L, and R92Q) (Tardiff, 2005). The R92W mouse showed a severe biatrial dilation since birth while the R92L had normal atrial dimensions with a late-onset atrial dilation at 1 year of age. In the R92Q mutants (transgenic mouse lines expressing 30, 67, and 92% of their total cTnT protein as the R92Q mutant), atria were dilated at early disease stages and, importantly, atrial dimensions increased proportionally with the level of transgenic protein expression (Tardiff et al., 1999). By employing the 67% R92Q mutant, we recently showed that atrial dilation can be prevented in the R92Q mice, if treated with ranolazine since birth (Coppini et al., 2017).

In the present work 1) we described the association of individual *TNNT2* mutations with AF by revising the clinical atrial data from 45 *TNNT2*-HCM index patients 2) to clarify the cellular basis of atrial pro-arrhythmogenic substrate we studied the energetics, mechanics and contractility of atrial myocardium from two well known HCM mouse models expressing *TNNT2* mutations (R92Q and E163R) selected because of their likely different association to AF. In our patient cohort R92Q is a hot spot mutation whereas E163R (like the ΔE163 in our cohort) is much likely a sporadic mutation. The combined clinical and biophysical analysis presented in this work supports the hypothesis that mutations in specific *TNNT2* hot-spots are more frequently associated with AF, triggered by intrinsic cellular mechanisms, than sporadic *TNNT2* mutations. The difference may affect the individual response to different treatment options.

## Methods

### Patients with *TNNT2* Mutations: Genetic Testing and Atrial Phenotype

Patient data collection is as described in Coppini et al., 2014. In brief, after informed consent, patients were screened for mutations in protein-coding exons and splice sites of 8 myofilament genes, including the thin-filament genes *TNNT2*, *TNNI3*, *TPM1*, and *ACTC*; the thick-filament genes *MYBPC3*, *MYH*7, *MYL2,* and *MYL3*. Genetic testing using established methods available at screening was performed by Clinical Laboratory Improvement Amendments (CLIA)–certified laboratories in the United States and at the Genetics Unit of the Careggi University Hospital in Florence. Presence of AF (either proximal and sustained) and Non Sustained Ventricular Tachycardia (NSVT) were evaluated by ECG analysis. Atrial dimensions and additional echocardiographic parameters were assessed using commercially available instruments.

### TnT Mutant Transgenic Mouse Lines

All experimental protocols on mice were performed in agreement with current Italian and European regulations and were approved by the local institutional review board and by the animal-welfare committee of the Italian Ministry of Health. We used a total of 27 6-to-8 month-old male C57BL/6N transgenic mice carrying the R92Q (Tardiff et al., 1999; Javadpour et al., 2003) or the E163R (Moore et al., 2014) mutation in the *TNNT2* gene, as well as Wild-Type (WT) littermates: 8 R92Q, 9 E163R and 10 WT mice were used for the experiments described below. The mouse colonies were housed in the animal facility of the University of Florence and all experiments were conducted locally. Transgenic protein expression levels of the R92Q and E163R lines were determined in the whole heart to be 67 and 50% mutant forms, respectively (Tardiff et al., 1999; Moore et al., 2014).

### Echocardiography

Echocardiographic studies were performed on isoflurane-anesthetized mice as previously described (Pistner et al., 2010) to characterize left-atrium (LA) morphology and LV diastolic function, using a Vevo 2,100 small animal echocardiography setup (Fujifilm Visualsonics). In brief, mice were anesthetised with 1.5% isoflurane and imaged in the supine position using a Vevo 2,100 Imaging System with a 40-MHz linear probe (Visualsonics, Canada). Core temperature was maintained at 37°C. Heart rates were kept consistent between experimental groups (400–500 bpm). ECG monitoring was obtained using limb electrodes. A standard 2D echocardiographic study was initially performed in the parasternal long-axis view for assessment of LV dimensions and LA diameter. The maximal anteroposterior LA diameter was measured using the M-mode. Doppler flow profiles were acquired using pulsed wave Doppler in the apical 4-chamber view. The sample volume was placed close to the tip of the mitral leaflets in the mitral orifice parallel to the blood flow in order to record maximal transmitral flow velocities. To assess the Isovolumic Relaxation Time (IVRT), a simultaneous mitral inflow and aortic outflow profile was recorded, allowing measurement of the time interval between aortic valve closure and mitral valve opening. Left atrial area was quantified in the apical 4-chamber view by tracing the border of the left atrium. Measurement was performed in end-systole before opening of the mitral valve.

### Mechanics and Energetics of LA Trabeculae

To prepare *intact atrial trabeculae*, we used methods solutions and protocols previously described for intact ventricular trabeculae (Ferrantini et al., 2014; Ferrantini et al., 2015). Briefly, mice were heparinized (5000 UI/ml) and anesthetized by inhaled isoflurane. The heart was rapidly excised, perfused retrogradely via the proximal aorta with a modified Krebs-Henseleit solution and placed in a dissection dish. The LA was opened and thin unbranched trabeculae were dissected by removing a portion of the LA wall on both ends. LA intact trabeculae were mounted between the basket-shaped platinum end of a force transducer and a motor, both connected to micromanipulators, in a glass-bottomed heated horizontal tissue bath with platinum wires for field stimulation. Sarcomere length was measured by laser5 diffraction. A custom-made Labview software was used for motor control, stimulation and force and length signal recording. Muscles were allowed to stabilize under our control conditions (30°C, 2 mM extracellular [Ca^2+^]], 1 Hz stimulation frequency) and were gradually stretched to optimal initial sarcomere length (2.15 ± 0.03 µm) before starting the experimental protocol. LA intact trabeculae were used to record isometric force during electrical stimulation with different pacing protocols and experimental conditions (Ferrantini et al., 2014; Crocini et al., 2016; Ferrantini et al., 2017). The cross-sectional area of the trabecula was calculated with the assumption of an ellipsoid shape.

LA trabeculae, dissected as described above, were permeabilized by overnight incubation in relaxing solution added with 0.5% Triton X100. Triton was then removed and the *atrial permeabilized trabeculae* were mounted horizontally between a force transducer and a motor by means of T-clips. The length of the preparations was adjusted to a sarcomere length of ∼2.15 µm. Tension-pCa curves (where pCa is equal to −log_10_ [Ca^2+^]) were obtained as previously described (Chandra et al., 2001). Muscles were activated by transferring them manually between baths containing different pCa solutions and the pCa-tension relationship was determined. The tension-pCa data were fit using the equation, P/Po = 1/[1 + 10^n^ x (pCa_50_-pCa)], where P is tension Po is maximum tension at saturating [Ca^2+^], n is the Hill coefficient, and pCa_50_ is equal to −log_10_ [Ca^2+^] required for producing half-maximal tension.

Sarcomere energetics was assessed in permeabilized trabeculae by simultaneous measurement of isometric tension and ATPase activity with an enzyme-coupled assay. The experimental procedures, solutions, and equipment used were as described previously (Witjas-Paalberends et al., 2014a; Witjas-Paalberends et al., 2014b). Isometric force and ATPase activity were measured at maximal and submaximal [Ca^2+^] at 20°C. The Ca^2+^-activated ATPase activity was calculated by subtracting the basal ATPase activity (measured in relaxing solution) from the total ATPase activity measured during contraction and normalized to the volume of the trabecula. LA trabecula volume was calculated as the sum of a cylinder (central body) and two elliptical cones (side) (see Figure 3A). The tension cost (energy cost of tension generation) was determined either from the ratio between ATPase activity and tension measured at maximal Ca^2+^ activation or as the slope of the relationship between ATPase activity and active tension measured at various pCa’s.

### Statistical Analysis

Unpaired Student t tests were used to compare normally distributed data from patients with different *TNNT2* mutations. Chi-square test was used to compare non-continuous variables expressed as proportions (Coppini et al., 2017).

Data from the mouse models are expressed as mean ± SEM (number of preparations and animals are indicated in the respective Figure legends). Statistical analysis was performed as previously described (Coppini et al., 2013; Coppini et al., 2017), using SPSS 23.0 (IBM, United States) and STATA 12.0 (StataCorp, United States). The three different groups (WT, R92Q, and E163R) were compared using One-Way ANOVA with Tukey correction (for normally-distributed homoscedastic datasets).

Overall, *p* < 0.05 was considered statistically significant. The range of calculated *p* values for each comparison (0.05 > *p* > 0.01, 0.01 > *p* > 0.001 or *p* < 0.001) is indicated in the respective figure panels using symbols: red symbols refer to R92Q vs. WT comparisons, blue symbols to E163R vs. WT comparison, and purple symbols to R92Q vs. E163R comparison.

## Results

### Different Association of Individual *TNNT2* Mutations With AF in HCM Patients

**FIGURE 1:**
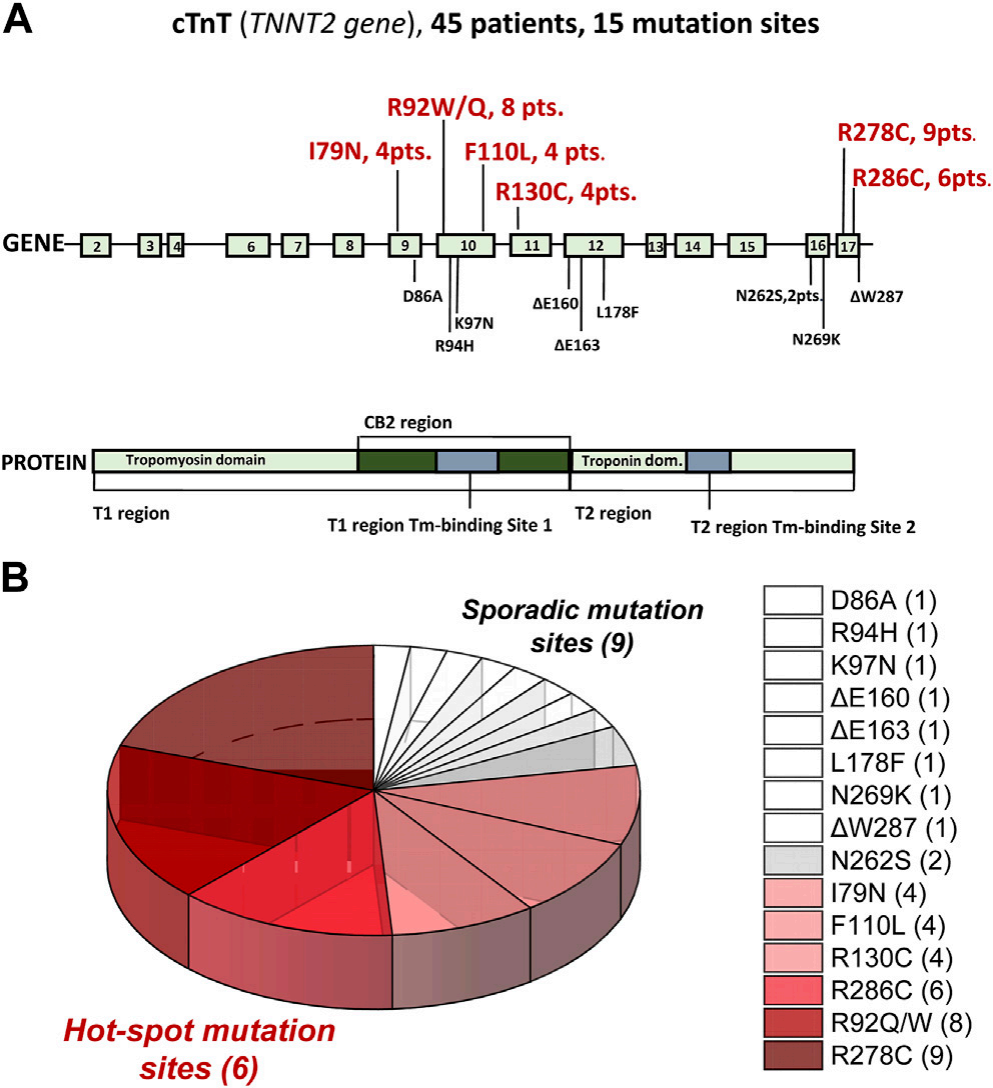
Sites and carrier numerosity of individual mutations in 45 *TNNT2*-positive Hypertrophic CardioMyopathy patients (HCM). **(A)** Representation of *TNNT2* exons (top panel) and structural and functional cTnT protein domains (bottom panel) showing hot (big red bold numbers) and sporadic (small black numbers) mutation sites. For mutations carried by more than one patient, the total number of carriers is indicated. **(B)** Pie chart of data reported in A: Six hot-spot mutation sites and nine sporadic ones are identified. Data modified and updated from Coppini et al., 2014.

Here, 45 cTnT-HCM patients, mostly those recruited in (Coppini et al., 2014) are reanalysed to assess the association of specific *TNNT2* mutations with AF (Figure 1 and Table 1). We indeed identified 10 patients with 9 mutations (D86A, R94H, K97N, ΔE160, ΔE163, L178F, N262S, N269K, and ΔW287) that are sparse in the *TNNT2* gene and “unexpectedly” sporadic (only 1/2 patients per each mutation among the 45 was found). They overall represent less than one fourth of the entire cTnT-HCM group (Figure 1). Of note, the prevalence of some mutations (ΔE160, ΔE163) appears rather low compared to previous description (Watkins et al., 1995) likely because we confined the analysis to index patients. The remaining 35 cTnT-HCM patients are carriers of mutations clustered in six “hot-spot” sites: I79N, R92Q/W, F110L, R130C, R278C, and R286C. For each mutation, 4 to 9 index patients were identified (Figure 1; Table 1).

**TABLE 1:** *TNNT2* sporadic and hot spot mutations associated to Hypertrophic CardioMyopathy (HCM) and prevalence of arrhythmias and clinical phenotype.

cTnT Mutations	N° of patients	Positive Arrhythmic burden	AF	NSVT	AF and NSVT	Atrial dilation	Final NYHA class III or IV
***Sporadic sites***							
D86A (1)	1	0	0	0	0	0	1
R94H (1)	1	0	0	0	0	1	1
K97N (1)	1	0	0	0	0	0	0
ΔE160 (1)	1	0	0	0	0	0	2
ΔE163 (1)	1	0	0	0	0	0	1
L178F (1)	1	0	0	0	0	0	1
N262S (2)	2	0	0	0	0	0	1
N269K (1)	1	0	0	0	0	0	1
ΔW287 (1)	1	1	1	0	0	0	1
Total	10	1	1	0	0	1	9
***Hot-spot sites***							
I79N (4)	4	2	2	2	2	2	2
R92Q/W (8)	8	3	2	2	1	2	0
F110L (4)	4	2	1	2	1	1	1
R130C (4)	4	3	3	1	1	2	1
R278C (9)	9	4	3	3	2	3	2
R286C (6)	6	3	2	2	1	2	1
Total	35	17	13	12	8	12	7
**p(Chi-square)**		**0.0281**	0.1020	**0.0306**	0.0955	0.1351	**0.0001**

AF, atrial fibrillation, NSVT, non-sustained ventricular tachycardia. Data modified and updated from Coppini et al., 2014.

The overall arrhythmic burden (quantified as the occurrence of any type of atrial or ventricular arrhythmia) was significantly higher in patients with mutations in the “hot-spot” sites (17 of 35) compared to those with mutations in the “sporadic” sites (1 of 10, *p* < 0.05, see table 1). By analysing separately atrial and ventricular episodes, we found that 1) among patients with mutations in the sporadic sites, none had NSVT episodes while NSVTs were described in 12/35 patients in the hot-spot group (*p* < 0.05), 2) AF was identified in only 1 out of 10 patients with sporadic mutations, while the occurrence of AF was much higher (from 25 to 75%) in patients with hot-spot mutations.

Interestingly, none of the patients in the sporadic group showed both AF and NSVT, while among the 35 with mutations in the hot-spot sites, eight were positive for both AF and NSVT, potentially unveiling a common mutation-driven substrate for arrhythmias in both cardiac chambers.

We selected the mutation site E163 (rarely associated to AF) and R92 (more frequently associated to AF) to investigate mutation-driven arrhythmogenic mechanisms of atrial myocardium in the R92Q and E163R mouse models that were available for *in vivo* and *in vitro* functional studies.

### Left Atrial Dilation Is More Severe in R92Q Compared to E163R Transgenic Mice

**FIGURE 2:**
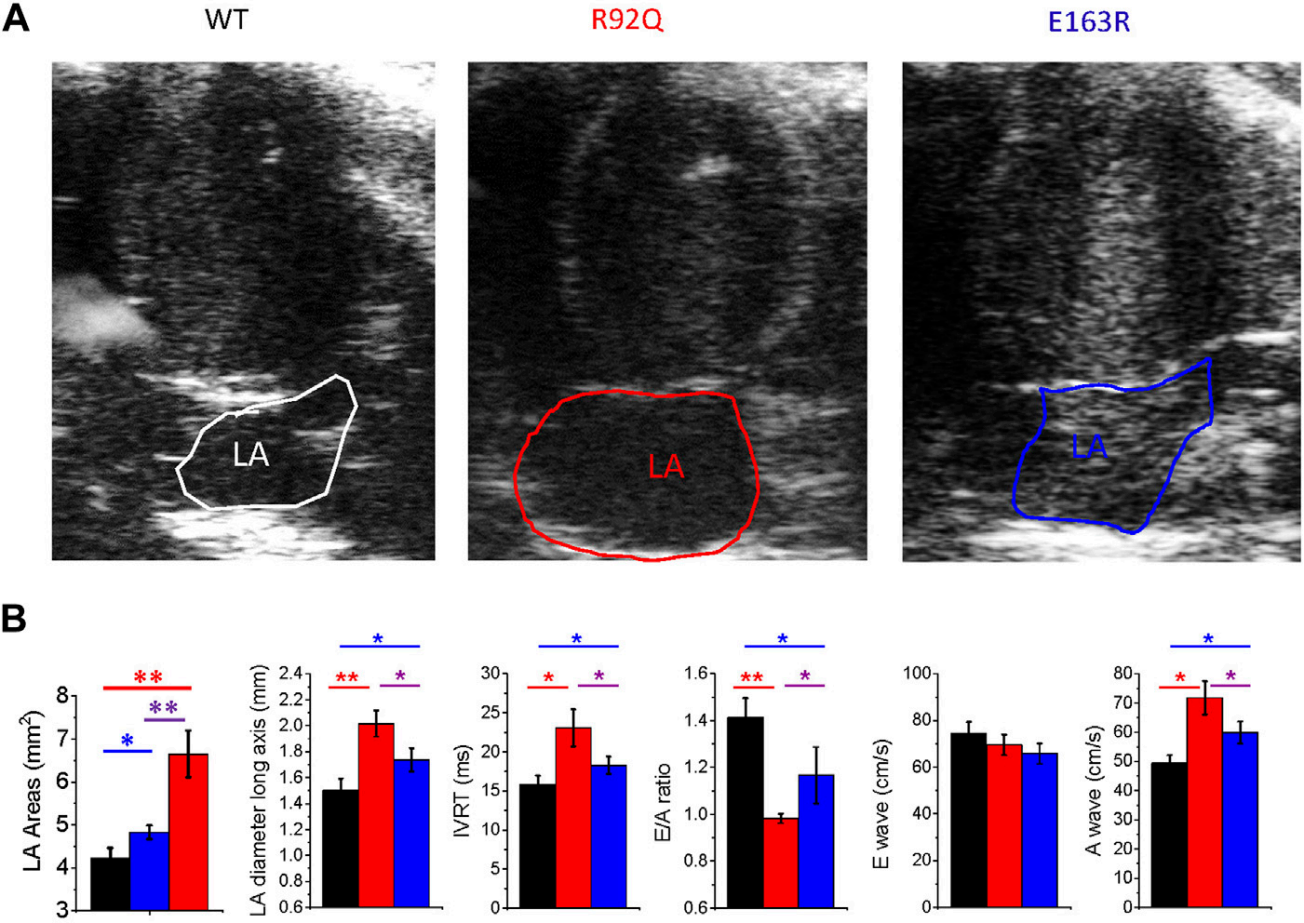
Echocardiographic measurements in WT and cTnT mutant mouse models. **(A)** Representative echocardiographic images of left atrial area (LA) of WT, R92Q and E163R mice. **(B)** Comparison of LA areas, maximal anteroposterior left atrial diameter measured from parasternal long axis view, ratio between E and A waves (E/A ratio), isovolumic relaxation time (IVRT), E-wave and A wave in mice from the three study cohorts. Statistical tests: One-way ANOVA with Tukey correction. Data are Means ± S.E.M. from 10 mice per group. * *=* 0.05*>p* > 0.01*;* ** = 0.01 > *p* > 0.001.

Echocardiographic measurements were performed in anesthetized male mice from the three study groups (WT, R92Q, and E163R), using a standardized protocol (Merx et al., 2014) and focussing on the atrial characteristics and LV diastolic function. Representative images of four-chamber long axis views are reported in Figure 2A. The highlighted perimeter of LA was used to estimate the LA circumferential area, while atrial anteroposterior diameter was measured in the parasternal long axis view (Figure 2B). In both R92Q and E163R, the LA areas and diameters were significantly increased compared to WT (Figure 2B), demonstrating the presence of atrial dilation in both HCM models. However, it is important to note that the extent of atrial dilation was minimal in the E163R (+15%) but markedly severe in the R92Q (+125%). This was paralleled by a markedly worse diastolic function in R92Q mice, when compared with E163 (Figure 2B), as previously observed (Ferrantini et al., 2018). Indeed, the isovolumic relaxation time (IVRT) was markedly increased and the E/A ratio decreased in the R92Q mice compared to WT while they were less affected in the E163R hearts. Interestingly, the marked decrease of the E/A ratio in the R92Q mice was mainly driven by a larger A wave, corresponding to transmitral flow during atrial contraction (see Figure 2B).

### Energy Cost of Tension Generation and Calcium Sensitivity of R92Q and E163R Atrial Myocardium: The Sarcomere Changes Are Similar to Those Observed in the Corresponding Ventricles

**FIGURE 3:**
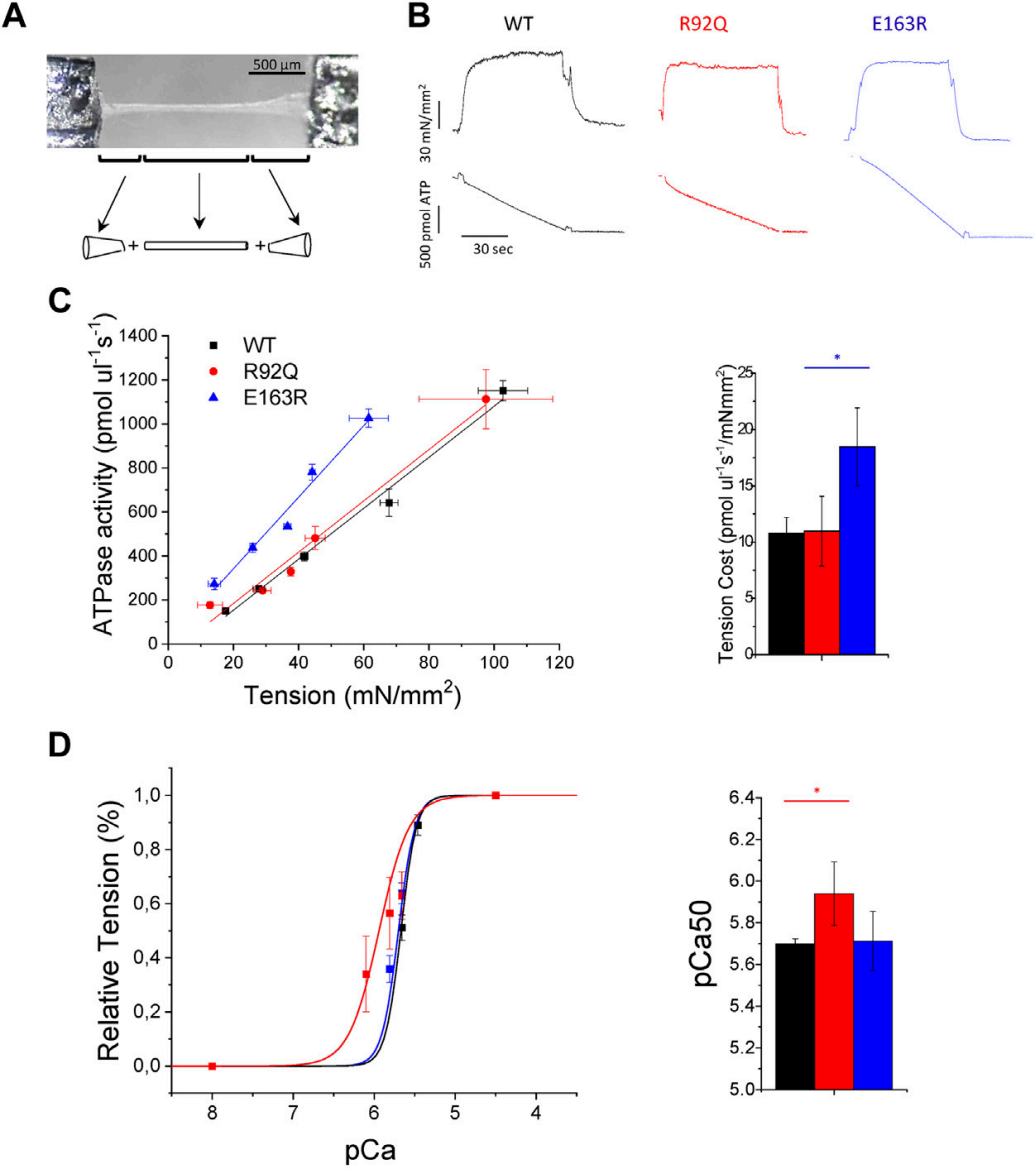
Mechanics and energetics of permeabilized atrial trabeculae from WT and cTnT mutant mouse models. **(A)** Picture of one permeabilized atrial trabecula with a scheme of the method used to calculate the volume of the trabecula assumed to be a cylinder between two elliptical cones **(B)** Representative tension (top) and ATPase activity (bottom) traces from wild-type (WT), R92Q, and E163R trabeculae that were maximally Ca^2+^-activated (pCa 4.5) at 20°C. Tension cost can be estimated as the ratio between the maximal Ca^2+^-activated ATPase activity and isometric tension (see Table 2). **(C)** Relationship between isometric tension and ATPase activity measured at different pCa’s in permeabilized atrial trabeculae from the three groups of mice. The tension cost can be measured from the slope of the ATPase-tension relationship; mean tension cost values are reported at the right. Mean ± SE of tension cost was calculated at 20°C. from WT (N = 6, *n* = 10), R92Q (N = 3, *n* = 6) and E163R (N = 4, n = 8) mice: N = number of animals, n = number of trabeculae. Statistical test see below **(D)** pCa-Tension curves (left) and mean values of pCa at half-maximal activation (pCa_50_, right) from WT (N = 5, n = 10), R92Q (N = 3, n = 7) and E163R (N = 4, n = 9) trabeculae. Statistical tests for all measurements: One-way ANOVA with Tukey correction. * = 0.05 > *p* > 0.01; ** = 0.01 > *p* > 0.001.

Direct demonstration of the energetic impact of E163R-cTnT and R92Q-cTnT, expressed at 50 and 67% respectively in atrial sarcomere, was obtained by simultaneously measuring isometric tension and ATPase activity in permeabilized LA trabeculae (Figure 3A) at 20°C. Representative recordings are reported in Figure 3B. Both cTnT mutated strains generated maximal Ca^2+^-activated tension similar to WT but the maximal ATPase activity was markedly increased in the E163R permeabilized LA trabeculae (Table 2). The ratio between maximal Ca^2+^-activated ATPase and active tension, that represents the energetic cost of tension generation, was markedly higher in the E163R trabeculae while it was comparable to WT in the R92Q trabeculae (Table 2). The different behaviour of the tension cost in the E163R atrial muscle compared to WT and R92Q was confirmed by measuring tension and ATPase activity at sub-maximal [Ca^2+^] (Figure 3C). Tension cost measured by the slope of the relation between Ca^2+^-activated ATPase activity and tension was significantly higher in the E163R compared to WT and R92Q (Figure 3C; Table 2). It is worth noting that increased tension cost has been associated with an increased cross-bridge detachment rate (Vitale et al., 2021; Ferrantini et al., 2017).

**TABLE 2:** Mechanical energetic and myofilament Ca^2+^-sensitivity parameters of permeabilized atrial trabeculae from WT, E163R, and R92Q mice.

	P_o_	ATPase_MAX_	ATPase_rest_	TC (slope)	TC (Max)	pCa_50_	n_H_
	(_mN/mm_^2^)	_(pmol ul_^−1^ s^−1^)	_(pmol ul_^−1^ s^−1^)	_(pmol ul_^−1^ s^−1^/_mN/mm_^2^)	_(pmol ul_^−1^ s^−1^/_mN/mm_^2^)		Cooperativity
WT (N = 7)	65.31 ± 6.22	655.89 ± 93.49	136.13 ± 11.02	10.779 ± 1.40	9.47 ± 1.39 (*n = 8*)	5.7 ± 0.02	4.23 ± 0,8 (*n = 10*)
	*(n = 10)*	*(n = 15)*	*(n = 10)*	*(n = 10)*		*(n = 10)*	
E163R (N = 2)	59.92 ± 6.75	771.91 ± 90.9* (*n = 8*)	189.47 ± 41.63	18.46 ± 3.47* (*n = 8*)	19.03 ± 4.5* (*n = 8*)	5.67 ± 0.14	4.54 ± 0.62 (*n = 7*)
	*(n = 8)*		*(n = 8)*			(n = 7)
R92Q (N = 2)	58.16 ± 12.3 (n = 8)	603.87 ± 187.3 (n = 7)	142.34 ± 30.10 (n = 7)	10.98 ± 3.10 (n = 7)	9.12 ± 4.48 (*n = 8*)	5.94 ± 0.15* (n = 7)	2.33 ± 1.05* (n = 7)

Tension cost (TC) data are measured from ATPase and isometric tension values measured at maximal activation (TC max) or from the slope of the ATPase tension relationship measured at different pCa’s (TC slope); see Figure 3. Po maximal isometric tension. pCa_50_, −log10 [Ca^2+^] required to get half maximal tension; n_H_, Hill coefficient. Statistical test: one-way ANOVA with Tukey correction (”*” indicates a statistically significant difference).

The average pCa-tension relationships obtained from the same permeabilized trabeculae showed a marked left shift in the R92Q preparations compared to both WT and E163R trabeculae (Figure 3D). The results in Figure 3D; Table 2 clearly show that myofilament Ca^2+^-sensitivity was significantly increased in the R92Q atrial trabeculae compared to WT while no difference was present in the E163R compared to WT mice. Interestingly, like in other cases of increased Ca^2+^ sensitivity in cardiac muscle (Kreutziger et al., 2011) the Hill coefficient (n_h_) of the pCa-tension relation is significantly decreased in the R92Q atrial trabeculae compared to both WT and E163R (Table 2).

Increased tension cost in the E163R atrial myocardium and increased myofilament Ca^2+^-sensitivity in the R92Q atrial myocardium are key alterations also identified in permeabilized ventricular trabeculae of the same mouse strains (Ferrantini et al., 2017). This suggests that these changes are most likely the direct, specific, consequences of the mutations that impact the sarcomere function of both cardiac chambers.

### Twitch Relaxation Is Prolonged and Spontaneous Activity Increased Only in R92Q Atrial Myocardium

**FIGURE 4:**
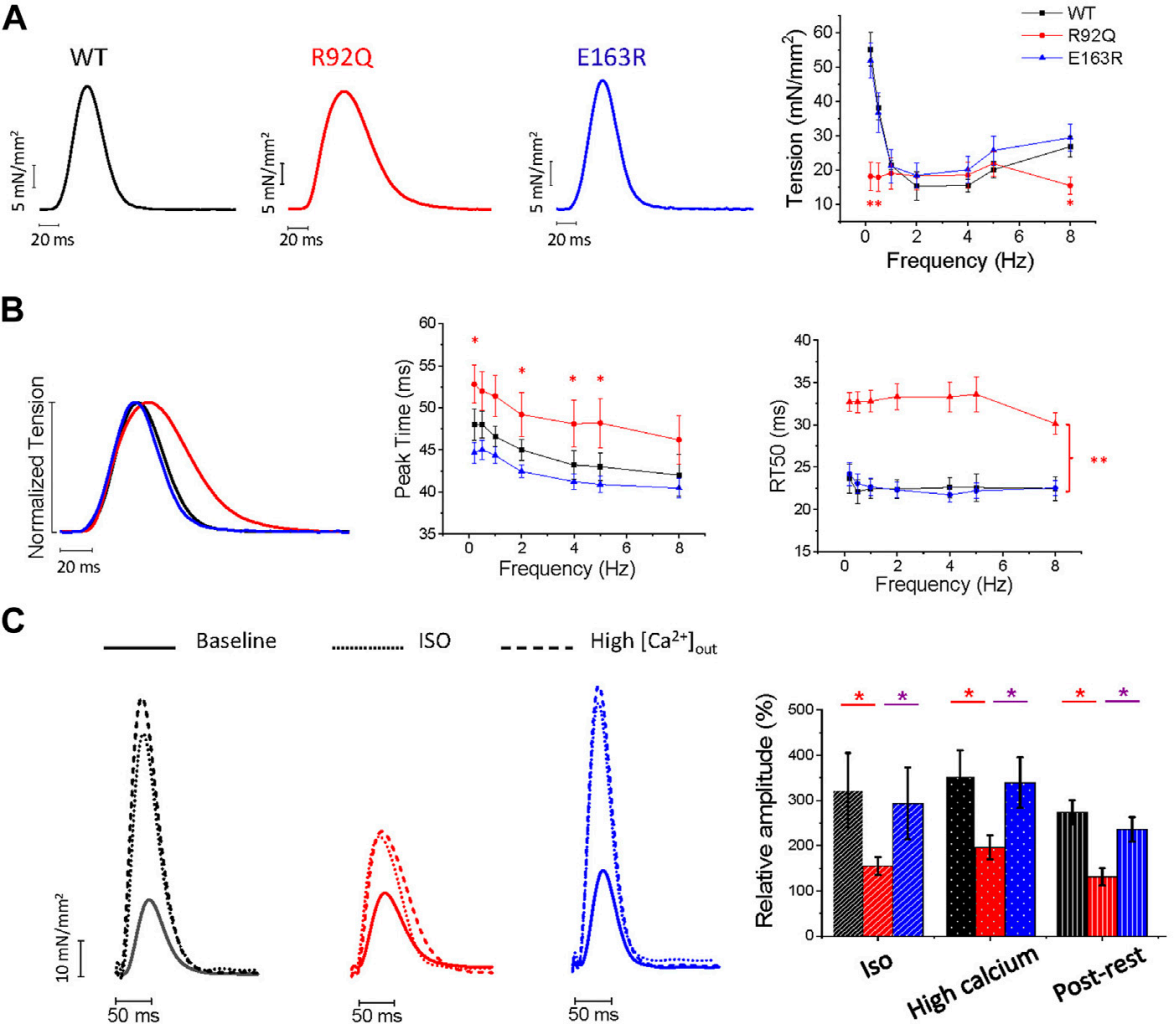
Comparison of isometric twitch amplitude and kinetics of intact atrial trabeculae from WT, R92Q and E163Rmice. **(A)** Left: representative tension traces of twitches from intact atrial trabeculae of WT, R92Q and E163R mice under control experimental conditions (30°C, 2 mM external [Ca^2+^], stimulation frequency 1 Hz). Right: relationship between stimulation frequency and peak isometric twitch tension in the three groups of intact atrial trabeculae. **(B)** Left: normalized superimposed twitches at 1 Hz stimulation frequency to highlight kinetics differences. Center: average time to peak at different stimulation frequencies in atrial trabeculae from the three groups. Right: average time from peak to 50% relaxation (RT_50_) in atrial trabeculae from the three groups. **(C)** Inotropic interventions in atrial trabeculae. Left: superimposed traces under control conditions and following addition of isoproterenol 1 µM (ISO) or 6 mM external [Ca^2+^] in trabeculae from WT, R92Q, and E163R mice. Right: variation of the peak twitch force from our control conditions following ISO, 6 mM external [Ca^2+^] and post rest potentiation. * = *p* < 0.05 ** = *p* < 0.01; One-way ANOVA with Tukey correction. WT (N = 9, *n* = 14), R92Q (N = 4, *n* = 10) and E163R (N = 5, *n* = 10) mice. N = number of animals, *n* = number of trabeculae.

Isometric force was measured from intact LA trabeculae during field-stimulation at 30°C under different stimulation protocols and various inotropic stimuli (i.e. Isoproterenol 1 µM (ISO), 6 mM extracellular [Ca^2+^] and post rest potentiation) (Figure 4). Under our control experimental condition, i.e. 1 Hz, 2 mM extracellular [Ca^2+^], the amplitude of twitch contractions was similar in WT, R92Q, and E163R trabeculae (Figure 4A). The plot relating twitch tension to stimulation frequency in Figure 4A shows that at low pacing frequency (0.5 Hz), R92Q atrial trabeculae developed lower twitch tension than E163R and WT. The overall force frequency relation seems to be blunted in the R92Q atrial trabeculae. In addition, the R92Q atrial trabeculae showed prolonged twitch duration compared to WT, due to a significant prolongation of both contraction and relaxation times (Figure 4B). No major variations of contraction kinetics, instead, were observed in the E163R preparations, although contraction peak time at low pacing rates was significantly faster in the E163R compared to WT trabeculae (Figure 4B).

To further evaluate the impact of the mutation on the mechanics of intact atrial trabeculae, we applied inotropic interventions (ISO, high extracellular [Ca^2+^], post rest potentiation) (Figure 4C). Under ISO, high extracellular [Ca^2+^] or post rest potentiation, R92Q preparations showed lower tension levels than WT indicating reduced inotropic response. Contrarily, E163R trabeculae showed a positive inotropic response under all inotropic interventions tested (Figure 4C) similar to that of WT atrial trabeculae.

**FIGURE 5:**
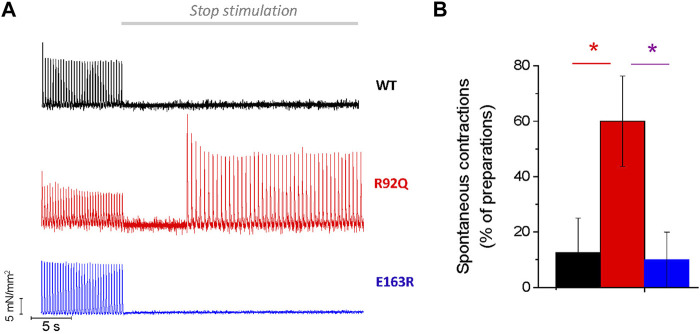
Spontaneous activity in R92Q atrial trabeculae. **(A)** Representative traces showing the stimulation protocol that may induce spontaneous activity upon stimulation pauses following a train of high frequency stimuli. R92Q but not E163R and WT trabeculae showed significant occurrence of spontaneous triggered activity during the resting pauses. **(B)** Percentage of trabeculae showing spontaneous activity during long pauses. One-way ANOVA with Tukey correction. * = *p* < 0.05. WT (N = 9, *n* = 14), R92Q (N = 4, *n* = 10) and E163R (N = 5, *n* = 10) mice. N = number of animals, *n* = number of trabeculae.

Finally, the arrhythmogenic propensity associated with each of the two mutations was assessed (Figure 5A). To increase the probability of spontaneous events, we applied a combination of ISO and a burst of high-rate (3 Hz) stimuli followed by a 30 s simulation pause. With this protocol, spontaneous activity occurred in more than 60% of the R92Q atrial trabeculae, while premature contractions occurred in less than 10% of the E163R and WT atrial trabeculae (Figure 5B).

## Discussion

The prevalence of AF in HCM was initially described to be independent from the underlying disease genotype. Olivotto et al., 2008 was the first report showing no difference between myofilament positive and myofilament negative patients in terms of AF prevalence (Olivotto et al., 2008). Furthermore, no differences were found between patients carrying mutations in thick or thin filament-related genes in more recent studies (Coppini et al., 2014; Bongini et al., 2016). However, in the thin filament subgroup, AF tends to onset at younger age (Bongini et al., 2016) and is more often aggressively treated with catheter ablation (Coppini et al., 2014). Indeed, in a recent prospective observational cohort study on more than 1,200 patients with early-onset AF (<66 years) (Yoneda et al., 2021), the cardiomyopathy-gene panel test identified a pathogenic sarcomere mutation in 10% of patients. This pivotal study supports the use of genetic testing in early-onset AF, although the clinical association between sarcomere mutation and AF remains controversial.

Approximately, 9–13% of HCM is caused by mutations in the *TNNT2* gene coding for cTnT (Coppini et al., 2017), a sarcomeric protein that anchors the cardiac ternary troponin (cTn) complex to tropomyosin (Tm) contributing to the modulation of calcium-induced activation of the cardiac thin filament. The cTnT protein comprises two functional domains: the Tm-binding T1 domain and the calcium-sensitive T2 domain, which binds cardiac troponin I (cTnI), cardiac troponin C (cTnC), and Tm (Gordon et al., 2000). A long (∼50-amino acids) flexible linker connecting the T1 and T2 domains (Figure 1A) is essential for transmitting the calcium-induced conformational changes from the C-terminal end of cTnT to the N terminus. Although cTnT is a relatively small protein (34.6 kDa), distinct regions have individual and specific functions. Thus, the molecular pathogenesis of HCM induced by mutations in different regions can be different and associated to different phenotypic manifestations in terms of degree of LV hypertrophy, diastolic dysfunction and propensity towards ventricular arrhythmias as well as, importantly, occurrence of AF and severity of atrial remodelling.

### Hot-Spot *TNNT2* Mutations Associated to High AF-Prevalence can be Identified in HCM Patients

Initial clinical phenotype descriptions of *TNNT2* mutations were from families with severe HCM (cTnT-HCM), characterized by mild hypertrophy and high incidence of sudden cardiac death (SCD). More recently the severity of cTnT-HCM has been reconsidered in light of large patient-populations (Coppini et al., 2014). Despite the occurrence of life-threatening ventricular arrhythmias that always catches the attention of clinicians and basic scientists, the most common sustained arrhythmia in HCM, including cTnT-HCM, is AF (Olivotto et al., 2001). Identification of hot-spot mutations in the *TNNT2* gene that are highly associated to AF is therefore potentially relevant to clinical decision-making, including risk stratification for AF prophylaxis.

In the present work we show that the association between *TNNT2* mutations and AF is variable.

In our cohort of HCM patients, we identified an association of individual *TNNT2* mutations with AF. AF was identified in 10% of patients with sporadic *TNNT2* mutations (D86A, R94H, K97N, ΔE160, ΔE163, L178F, N262S, N269K, and ΔW287), while AF occurrence was much higher (25–75%) in patients carrying specific “hot-spot” mutations (I79N, R92Q/W, F110L, R130C, R278C, and R286C). Interestingly, only in patients with hot-spot mutations we observed a positive association between AF and ventricular arrhythmias in the form of NSVT, that may unveil a common mutation-driven substrate. In fact, in both cardiac chambers, highly enhanced cellular automaticity can be the cause of arrythmia onset and recurrences, promoting both AF and NSVT.

### R92Q vs. E163R TNNT2 Mouse Models: Severity of Atrial Myopathy and Propensity Towards Arrhythmias Depend on Genotype

Two HCM mouse models with R92Q and E163R cTnT mutations were studied with *in vivo* echocardiography and *in vitro* biophysical measurements to assess the extent of LA remodeling and the relative roles of myofilament dysfunction and excitation-contraction coupling changes in HCM pathophysiology. This choice was related to the availability of two mouse models with mutations at sites corresponding to a human hot (92) or sporadic (163) spot in the *TNNT2* gene respectively.

In our previous study on ventricular myocardium we showed that both R92Q and E163R mouse models exhibited ventricular hypertrophy, with prolonged twitch contractions, and increased arrhythmogenicity (Ferrantini et al., 2017; Vitale et al., 2021). At the sarcomere level, the two mutant proteins lead to the same effects in the atria as observed in the ventricles. Indeed, in E163R atrial myocardium the energy cost of tension generation is increased, likely due to increased cross-bridge cycling rate (Ferrantini et al., 2017) (with no significant variation of calcium sensitivity), while in the R92Q atrial myocardium we observed a rather large increase in calcium sensitivity (with no variation of cross-bridge energetics and cycling rates).

LA systolic function is typically an auxotonic contraction with much lower after-load and much higher shortening velocity compared to the LV contraction. For this reason, increased isometric cross-bridge cycling rates and ATP consumption in the E163R atrial myocardium may not represent a significant disadvantage for the atrial chambers. Consistently, in E163R intact atrial trabeculae twitch contraction parameters are normal or even slightly faster at low pacing rates (peak time), likely reflecting faster cross-bridge cycling rate during force development. Because of the different working conditions of the LA with respect to the LV, determining lower ATP consumption per unit of myocardial mass, the increased tension cost of E163R atrial myocardium may not significantly affect global cardiomyocyte energy balance, at variance with ventricular tissue. Therefore, the E163R mutation in atrial tissue may be unable to activate the pathological signalling pathways of cardiomyocytes that are most likely responsible for the slower twitch duration and the increased propensity to arrhythmias observed in the ventricular myocardium. The most relevant signalling pathway involved is likely the Ca-calmodulin Kinase II (CaMKII) pathway. Indeed, increased CaMKII activation has been observed in the ventricular tissue of HCM patients (Coppini et al., 2013; Coppini et al., 2018) and is associated with the severity of disease presentation (Helms et al., 2016) and with the occurrence of arrhythmogenic remodelling (Coppini et al., 2017) of HCM ventricles. In E163R atria, however, the occurrence of spontaneous contractions was not different from that of WT. These results seem to exclude the idea that the E163R mutation itself, by changing cross-bridge cycling rates, may represent a direct trigger towards atrial arrhythmias. The mild increase of E163R atrial dimensions observed *in-vivo* may not be related to the mutation itself but, rather it may be related to hemodynamic factors and diastolic dysfunction, that explain the late-onset of atrial dilation, governed by the severity of the LV disease. Only at late stages of the disease, atrial dilation due to LV dysfunction can promote and sustain AF.

In R92Q atrial myocardium, instead, increased myofilament calcium sensitivity can directly impair atrial contraction and predispose towards arrhythmias, similarly to the ventricular myocardium (Ferrantini et al., 2017). Again, increased CaMKII activity due to diastolic Ca^2+^ accumulation in the cytosol (a direct consequence of increased myofilament Ca^2+^-sensitivity) may explain the increased arrhythmic propensity and the extended functional alterations observed in the R92Q atrial myocardium (Coppini et al., 2018). Twitch contractions are severely prolonged (particularly in the relaxation phase) and the occurrence of spontaneous beats (likely related to Delayed After Depolarizations (DADs)) is markedly increased. In R92Q ventricular myocytes, we previously found that altered Ca^2+^-transient kinetics and increased Ca^2+^-dependent arrhythmogenesis are linked with increased CaMKII activation (Coppini et al., 2017). Although we did not directly measure calcium fluxes or CaMKII activity in R92Q atrial myocardium, the results unveil an intrinsic sarcomere mutation-driven mechanism associated to a higher occurrence of arrhythmias in both cardiac chambers.

Previous studies on I79N, F110I, R278C *TNNT2* mutations (all hot-spot sites for AF, according to our analysis in HCM patients) clearly demonstrated that increased myofilament Ca^2+^-sensitivity, by modifying cytosolic Ca^2+^ buffering, promotes DADs and triggered activity (Schober et al., 2012), representing a direct cause of arrythmias in both atrial and ventricular cardiomyocytes. In agreement, crossing transgenic mice expressing pathogenic cTnT mutants with opposite effects on myofilament Ca^2+^ sensitivity attenuates cardiomyopathy phenotypes and propensity to arrythmias in mice (Dieseldorff Jones et al., 2019).

### Clinical and Therapeutical Implications

**FIGURE 6:**
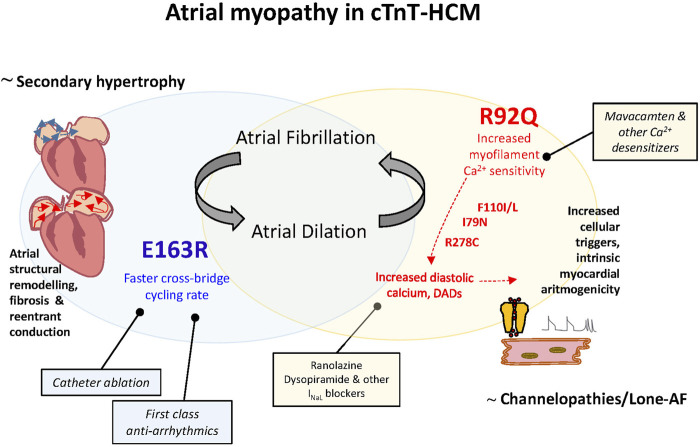
Atrial myopathy in cTnT-HCM: different pathogenesis of atrial cardiomyopathy, different genotype-driven therapeutical strategies for AF. Left: Atrial myopathy in E163R cTnT-HCM is likely a consequence of increased LV filling pressures as in secondary hypertrophy and other chronic LV conditions. In this case, AF would be predominantly sustained by atrial tissue structural remodeling and associated micro and macro reentrant conduction and first class antiarrhythmics to slow down conduction velocity and catheter ablation could be the recommended first choice treatments. Right: Atrial myopathy in R92Q cTnT-HCM is likely a direct effect of the mutation as in other genetic-based forms of AF. We identified the increased atrial and ventricular myofilament calcium sensitivity associated to this mutation as the trigger for cellular arrhythmias determined by calcium handling dysregulation, as previously demonstrated for other cTnT mutations. In this case drugs that directly decrease sarcomere calcium sensitivity or ameliorate cell calcium homeostasis can be more beneficial compared to classical antiarrhythmics and invasive treatments.

The principal role of the LA is to modulate LV filling and cardiovascular performance by operating as a reservoir for venous return during LV systole, a conduit for venous return during early LV diastole, and as a booster pump that augments LV filling during LV diastole. Thus, while LA compliance (or its inverse, stiffness) is the major determinant of reservoir function and conduit function, atrial booster-pump function reflects the magnitude and timing of atrial contraction, that occurs against a low after-load (<20 mmHg, compared to more than 100 mmHg in the ventricle) with a relatively large percentage change in chamber volume (>20–30). Here we demonstrate that increased isometric cross-bridge detachment rates and ATP consumption in the E163R atrial myocardium may not represent a significant disadvantage for the atrial chambers and is unlikely to initiate/promote E-C coupling alterations. In fact, twitch contraction amplitude and kinetics are preserved in E163R and no premature contractions were observed, suggesting no major changes in cardiomyocyte calcium handling. Indeed, in our E163R mouse model, atrial myopathy is manifested only as a mild dilation, likely a consequence of increased LV filling pressures due to LV diastolic dysfunction. This consideration in the murine model does not exclude that in selected patients carrying the E163R mutation, LV remodelling over time may lead to severe LV hypertrophy. In this case, as in secondary hypertrophy and other chronic LV conditions, AF may occur and would be predominantly sustained by tissue structural remodeling, fibrosis, micro and macro reentrant conduction (Figure 6). In this scenario, catheter ablation could be a recommended treatment.

According to the results reported here and in Ferrantini et al. (2017), increased cross bridge cycling rates and the energetic dysfunction associated with the E163R mutation affect contractility only when high-pressure generation is required (i.e. in the ventricles and not in the atria). As mentioned, we cannot exclude that -over timeprogressive LV remodelling trough mechanisms that are not specifically related to the mutation (e.g. fibrosis, myocardial disarray) causes LA remodeling, with increased LA pressures to levels that are critical for the E163R myocadium cross bridge cycling and energetic requirements.

On the contrary, atrial myopathy in R92Q cTnT-HCM is manifested as moderate-to-severe atrial dilation associated to atrial arrhythmias. In this case both the loss of atrial inotropic reserve (promoting dilation) and the membrane instability (premature contractions) are direct effects of the mutation. In this sense the R92Q cTnT-associated AF cannot be reconducted to mechanisms of secondary hypertrophy but rather resemble other genetic-based forms of AF associated to increased cellular arrithmogenicity (e.g. channelopathies, lone-AF (Figure 6). We identified the increased myofilament calcium sensitivity associated to R92Q cTnT mutation as the key trigger for calcium handling dysregulation, as previously demonstrated for other cTnT mutations and largely highlighted in R92Q cTnT ventricular myocardium (Ferrantini et al., 2017). From the therapeutical standpoint, we can hypothesize that, in the presence of these mutations, drugs that decrease sarcomere calcium sensitivity (including the novel first-in-class myosin inhibitors) or drugs that ameliorate cell calcium homeostasis can be more effective on atrial arrhythmogenesis compared to classical pharmacological approaches or invasive catheter treatments. We previously observed that ranolazine, by blocking late Na current, has the ability to normalize Ca-handling in human HCM myocardium (Coppini et al., 2013) and in the ventricles of R92Q mice (Coppini et al., 2017), and may thus reduce Ca-dependent arrhythmias in R92Q atria as well. Ranolazine also has the ability to selectively inhibit peak Na current in the atria destabilizing atrial reentry circuits (Burashnikov et al., 2007). These observations prompt towards further tests of ranolazine as a drug to prevent AF in selected HCM patients with high risk mutations.

### Limitations

To study the link between AF and *TNNT2* mutation, we used two mouse models (R92Q and E163R) that, based on our patient data, were not the best models to use. It may be difficult to accept that R92 mutations in cTnT are highly associated with AF when only 2 patients out of 8 (i.e. 25%) had AF and to generalize that patients with E163 mutations don’t do AF when we had only one patient, without AF, with a truncation mutation in the 163 site. According to the patient data, other mutations, like the R130C and the N262S (representative of the hot spot and sporadic spot mutations respectively), could have been better models of investigation. We used the R92Q and the E163R simply because of their availability and because in the patient study the cTnT sites 92 and 163 were associated to hot spot and sporadic mutations. This choice may have not been the best one but it was our only realistic possibility.

In the present study we did not perform long term ECG analysis by telemetry in conscious animals to detect or induce AF or other arrhythmias. This had been done for the R92Q model by Jimenez and Tardiff (2011) who demonstrated abnormalities in conduction, ventricular ectopy and a prolonged P duration after isoproterenol injections. The conclusion that the increased myofilament calcium sensitivity associated to *TNNT2* hot spot mutations is the key trigger for the calcium handling dysregulation that promotes arrhythmias may be limited by the difficulties of relating *in vivo* patient (and animal) propensity to arrhythmias to *in vitro* data that measure the propensity to spontaneous activity.

It may have not been sound to conclude that the differences in atrial phenotype associated to hot spot and sporadic *TNNT2* mutations are due to different biophysical mechanisms based upon just the data from a single mouse model (likely not the best one) for each category of mutation. There is, however, a wealth of biophysical data in the literature examining the effects of various cTnT mutations in different experimental systems. Some of these data can actually be used to support the argument of our study. For instance, Chandra et al. (2005) found that different mutations (R92 W/L) at the 92 site, that we classified as a hot spot site, markedly increased calcium sensitivity while a truncation mutation (delta E160) in a site, that we classified as a sporadic site, increased myofilament calcium sensitivity only very slightly while it specifically increased sarcomere energetics and cross bridge cycling rates. These findings and additional reports confirming that a marked increase of myofilament calcium sensitivity occurs in all the hot spot mutations that have been investigated so far (at least to our knowledge, e.g. Miller et al., 2001 for the I79N; Groen et al., 2020 for the R130C; Schuldt et al., 2021 for the R278C) support the conclusion of our study.

Contractile function of cardiomyocytes depends on isoform types of key sarcomeric proteins (e.g., alpha/beta-MyHC, MLC-1a/v, MLC-2a/v) and the phosphorylation status of some of them (e.g., cTnT, cTnI, MLC-2, cMyBP-C). In the present study the potential contribution of differences in sarcomeric protein isoforms and phosphorylation levels to the mouse model phenotypes has not been investigated. Therefore, we cannot rule out that isoform shifts and post-translational changes in some of the sarcomeric proteins may have been involved in the changes of myofilament calcium sensitivity and/or cross bridge cycling rates found in this study. Additional work is needed to address this point.

## Conclusion

Overall, this work sets the stage for a reconsideration of patient genotype as a criterion for predicting AF in HCM patients. We suggest that the correlation is not gene-specific, not even mutation-specific, but rather “biophysical mechanism”-specific. Mutations that affect cross-bridge cycling are likely to be “E163R”-like (that do not directly promote AF and loss of atrial contractility), while mutations affecting myofilament calcium sensitivity are likely to be “R92Q”-like (that directly promote cellular arrhyhtmogeneicity). Moreover, if the association of *TNNT2* mutations with AF occurrence depends on the underlying mutation-driven pathomechanisms, then tailored preventive treatments for AF can be identified (including Mavacamten and Ranolazine) and tested in selected genotyped HCM subgroups (“R92Q”-like).

## Data Availability

The raw data supporting the conclusion of this article will be made available by the authors, without undue reservation.
